# Lemonade From Lemons: Recruiting Blood Stem Cells into Action

**DOI:** 10.1097/HS9.0000000000000416

**Published:** 2020-06-03

**Authors:** David G. Kent

**Affiliations:** York Biomedical Research Institute, Department of Biology, University of York, York, UK

The old saying says, “When life gives you lemons, make lemonade” and when it comes to laboratory experiments, researchers are typically given an awful lot of lemons. One of the research areas that have suffered through more than its fair share of frustrating experiments is human blood stem cell expansion where large numbers of failed attempts to expand stem cells for their use in transplantation medicine have been recorded over the decades. Mouse blood stem cell expansion on the other hand had a landmark paper^1^ last year reporting > 200-fold expansion of HSCs over a 1 month culture period. This has spurred a renewed to begin to achieve the same sorts of numbers in human expansion cultures, making it a very exciting area of science at present.

For immunological reasons, a preferred source of blood stem cells is cord blood, but cord blood has some limitations when it comes to graft success, since the number of cells is finite. Whereas cord blood transplantations are relatively routine in young children where the number of blood stem cells per cord blood is often sufficient to achieve donor cell engraftment, this is not the case for all adults where larger cell numbers are often in excess of what can be obtained from a single cord (one of the field's early pioneers, Hal Broxmeyer summarized the field quite nicely in this review^2^).

The mechanistic basis for this comes from the belief that the number of blood stem cells required for a patient is in direct proportion to their weight, meaning that larger patients require much higher doses of cord blood cells to obtain a sufficient number of stem cells. Dual cord blood transplantations have been explored^2^ to see if simply adding in more cells would be the answer, but dual cord transplantations have their own complications as the blood cells fight to establish themselves in the new environment (N.B. this is a fascinating process and not particularly well-understood from a mechanistic point of view). Moreover, clinical evidence suggests^3^ that alloreactivity can be increased by having two cord blood sources (as opposed to one) transplanted into a recipient. So, in the absence of enough stem cells in a single cord, options to increase numbers are limited, hence the incredible amount of research time and money invested in trying to expand the stem cell number in cord bloods prior to transplantation.

Curiously, we do not often discuss what it means to have “increased blood stem cells” in a pot of cells and as with nearly everything in the field of blood stem cells, the assays to demonstrate functionality are retrospective and consequently highly reliant on the permissiveness of the assay. It may, therefore, be just as important to ask the question of whether there are more “potential stem cells” in a cord blood than the ones that read out in a transplantation setting. If they were indeed found to be present, how might they be engaged to be productive in a transplantation scenario? This is exactly what Gupta et al., investigated in their recent paper entitled “Nov/CCN3 enhances cord blood engraftment by rapidly recruiting latent human stem cell activity”^4^ which was an exciting read. The study began with the hypothesis that some fraction of blood stem cells was present in a cord blood, but ineffective in transplantation because they were not appropriately prepared to engraft in a host. They had done a fair amount of previous work to identify the candidate NOV as a potential modulator of blood stem cells and this paper really focused on testing its ability to improve the frequency of cells able to engraft (rather than to expand them through self-renewal expansion divisions). Conceptually this is a really neat idea and suggests that priming stem cells for transplantation may be worth investing considerably more energy into.

Perhaps the most compelling experiment was a head-to-head comparison of CD34^+^ cells treated for 8 hours with NOV or not. Following rigorous limiting dilution primary and secondary transplantation experiments in mice, it was clear that the NOV-treated cells were superior in their ability to perform as functional stem cells in transplantation. Importantly, no cells divide in this short 8-hour culture, thereby excluding in vitro HSC expansion as a mechanism. This latter point was supported by in vitro single cell assays and the paper ends up concluding that coaxing cells to be in the correct “state” for subsequent engraftment could be achieved.

Previous studies have hinted that cell cycle regulation and “quiescence exit” in particular would be critical to our ability to manipulate how blood stem cells are called into action. CDK6 levels were identified by the Dick^5^ and Sexl groups^6^ in 2015 as a key regulator of quiescence exit and understanding its molecular mechanism would help determine the speed at which a blood stem cell could leave its hibernating state. Other studies have focused on the initial harvest of cord blood from donors. Of interest here is the work of Mantel/O’Leary et al^7^ who showed that reducing the loss of stem cells in a cord blood harvest could be achieved by not exposing them to extra-physiological oxygen levels.

Together, these multiple lines of evidence suggest that the potential for large numbers of stem cells exist in a single cord and it is down to the research community to identify the best method of preserving and/or activating them to be most productive in a post-transplantation scenario where they must seed the production of blood cells for the remainder of the patient's lifetime.
